# Targeting inflammasome pathway towards therapeutics of neurodegenerative diseases

**DOI:** 10.61747/0ifp.202503001

**Published:** 2025-04-28

**Authors:** Wanli Smith, Mali Jiang, Jazlynn Meza, Gabriela Mercado

**Affiliations:** 1 Department of Psychiatry, Division of Neurobiology, Johns Hopkins University School of Medicine, Baltimore, Maryland, USA 21287; 2 Department of Pharmacology, Johns Hopkins University School of Medicine, Baltimore, Maryland, USA 21287

**Keywords:** Inflammasome, neuroinflammation, neurodegeneration, NLRP3, Caspase-1

## Abstract

Inflammasome, an intracellular protein complex, is a crucial regulator of the innate immune response defending against harmful insults. Increasing evidence indicates that activation of inflammasome pathway in brain cells plays a significant pathological role in neurodegenerative diseases. Targeting this pathway offers a promising strategy for developing novel treatments. Here, we review the wide-ranging involvement of inflammasome activation in neurodegenerative diseases and highlight current therapeutic strategies targeting this critical pathway.

## INTRODUCTION

Neurodegenerative diseases represent a major healthcare crisis due to their increasing prevalence and the substantial burden they place on individuals, families, and healthcare systems. These diseases share common pathological hallmarks, including progressive neuronal loss leading to cognitive, motor or mood dysfunction, and the presence of intracellular/extracellular abnormal protein aggregates. While the etiology and molecular mechanisms remain incompletely understood, chronic neuroinflammation is believed to be a critical driver of disease development and progression. Recent studies have identified that activation of the inflammasome pathway in the central nervous system (CNS) as a potential contributor to neuroinflammation, neurodegeneration and disease onset and progression [1,2]. Therefore, modulating the inflammasome pathway may provide novel strategies to slow or delay disease progression. This review summarizes the broad involvement of inflammasome pathway activation in neurodegenerative diseases and highlights the current therapeutic approaches targeting this pathway.

### 1. DYSREGULATION OF INFLAMMASOME PATHWAY AND ITS LINK TO NEUROINFLAMMATION

#### 1.1. Neuroinflammation

A key pathological feature of neurodegenerative diseases, can act as either a trigger or a secondary response to neurodegeneration. Patients and animal models of these diseases display increased inflammatory responses, including microglia activation and elevated levels of inflammatory mediators (e.g., tumor necrosis factors (TNFs), caspase-1, and interleukins) in the brain, cerebrospinal fluid (CSF), and blood [3,4]. Microglia and astrocytes, resident CNS immune cells, interact with adaptive immune cells (T and B lymphocytes) in response to infections and non-infectious stimuli, resulting in inflammation-driven neurotoxicity. While the precise mechanisms underlying this inflammation remain poorly defined, recent studies suggest that inflammasome pathway activation in the CNS contributes to both neuroinflammation and neurodegeneration [1,2].

#### 1.2. Inflammasome

A multimeric protein complex, is a key component of the innate immune response. Its formation is triggered by substances arising from infections, tissue damage, or metabolic disorders (Table 1). A typical inflammasome comprises an NLRP (Nucleotide-binding oligomerization domain, Leucine rich Repeat and Pyrin domain containing) sensor molecule, the adaptor protein, apoptosis-associated speck-like protein (ASC) containing caspase recruitment domain (CARD), and inflammatory caspases such as caspase-1 [1]. Various internal (e.g., tissue damage debris) and external (e.g., bacterial toxins) stimuli can trigger inflammasomes formation through pattern recognition receptors (PRRs) [5,6]. PRRs recognized both pathogen-associated molecular patterns (PAMPs) and damage-associated molecular patterns (DAMPs) [5]. Common PRRs involved in inflammasome formation include Nod-like receptors (NLRs), absent in melanoma 2-like receptors (ALRs), and Toll-like receptors (TLRs). Well characterized inflammasomes include the NLR family of inflammasomes (NLRP1 [7,8], NLRP2 [9,10], NLRP3 [11], NLRP6 [12], NLRP7 [13], NLRP12 [14]), CARD-containing protein 4 (NLRC 4)-linked inflammasomes [15,16]), ALR family inflammasomes (AIM2)[17], interferon-γ-inducible protein 16 (IFI16) [18,19] and pyrin-linked inflammasomes [20–22].

Different stimuli activate specific inflammasomes. Bacterial toxins induce NLRP1; adenosine triphosphate (ATP) induces NLRP2 [9,10]; PAMPs and DAMPs induce NLRP3 [11], NLRP6 [12], NLRP7 [13], and NLRP12 [14]; and double-stranded DNA induces AIM2 [17] and IFI16 [19]. NLRC4 is activated by bacterial flagellin and/or the type III secretion system of bacterial pathogens; and pyrin recognizes RhoA inactivation by toxins and effector proteins [7,8,15,16,20–22].

**Figure 1.**
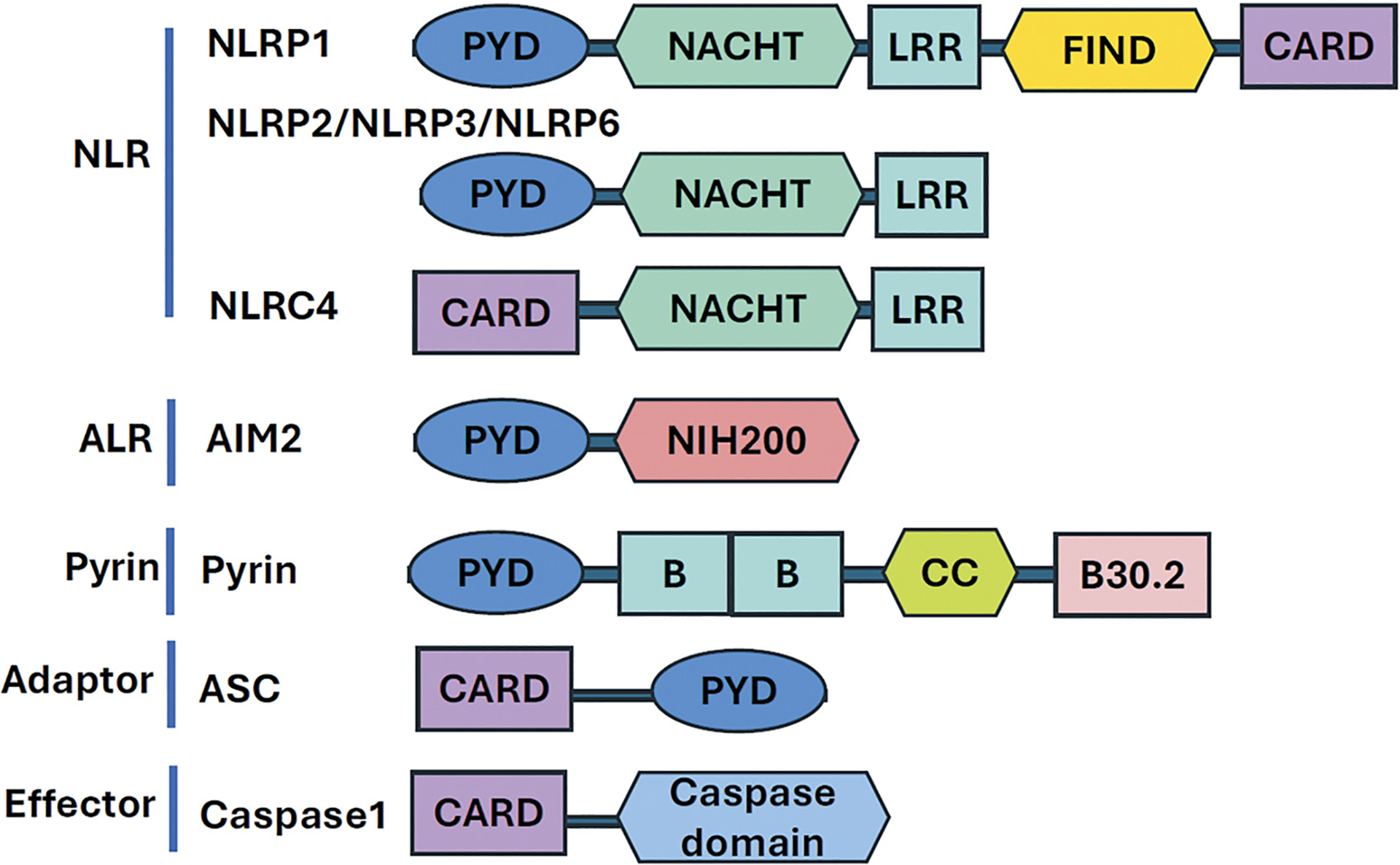
Inflammasome Components and Structure NLR and ALR receptor proteins recruit adaptor (ASC) and effector (caspase-1) proteins to form inflammasomes. NLRPs contain a nucleotide-binding and oligomerization domain (NACHT/NBD), and a leucine-rich repeat (LRR) motif. The NACHT domain is flanked by either CARD or PYD domain. AIM2, an ALR family receptor, has an amino-terminal PYD domain and one or two DNA-binding NIH200 domains. Pyrin (TRIM20) harbors a PYD domain, two B-boxes, a coiled-coil domain, and a C-terminal B30.2 domain. ASC, an adaptor protein for many types of inflammasomes, contains a CARD and a PYD domain, enabling homotypic interaction with PYD-containing inflammasome sensors (e.g., NLRP3, AIM2). Pro-caspase-1 has a CARD domain and a caspase domain, interacts with ASC via its CARD domain. This interaction leads to ASC oligomerization into a macromolecular aggregate (ASC speck) and pro-caspase-1 auto-cleavage into active caspase-1.

NLRP proteins are encoded by the NLR family genes, which comprises 14 genes in humans, a subfamily of the NOD-like receptor family of 22 genes in humans [5]. NLRPs contain a pyrin domain (PYD), a leucine-rich repeat (LRR) domain, a NACHT nucleotide-binding and protein oligomerization domain, and a caspase activation and recruitment domain (CARD) (Figure 1) [5]. NLRP1, NLRP2 and NLRP3 are highly expressed in neurons, astrocytes and microglia, respectively, and play critical roles in CNS inflammasome formation and activation [5].

**Figure 2.**
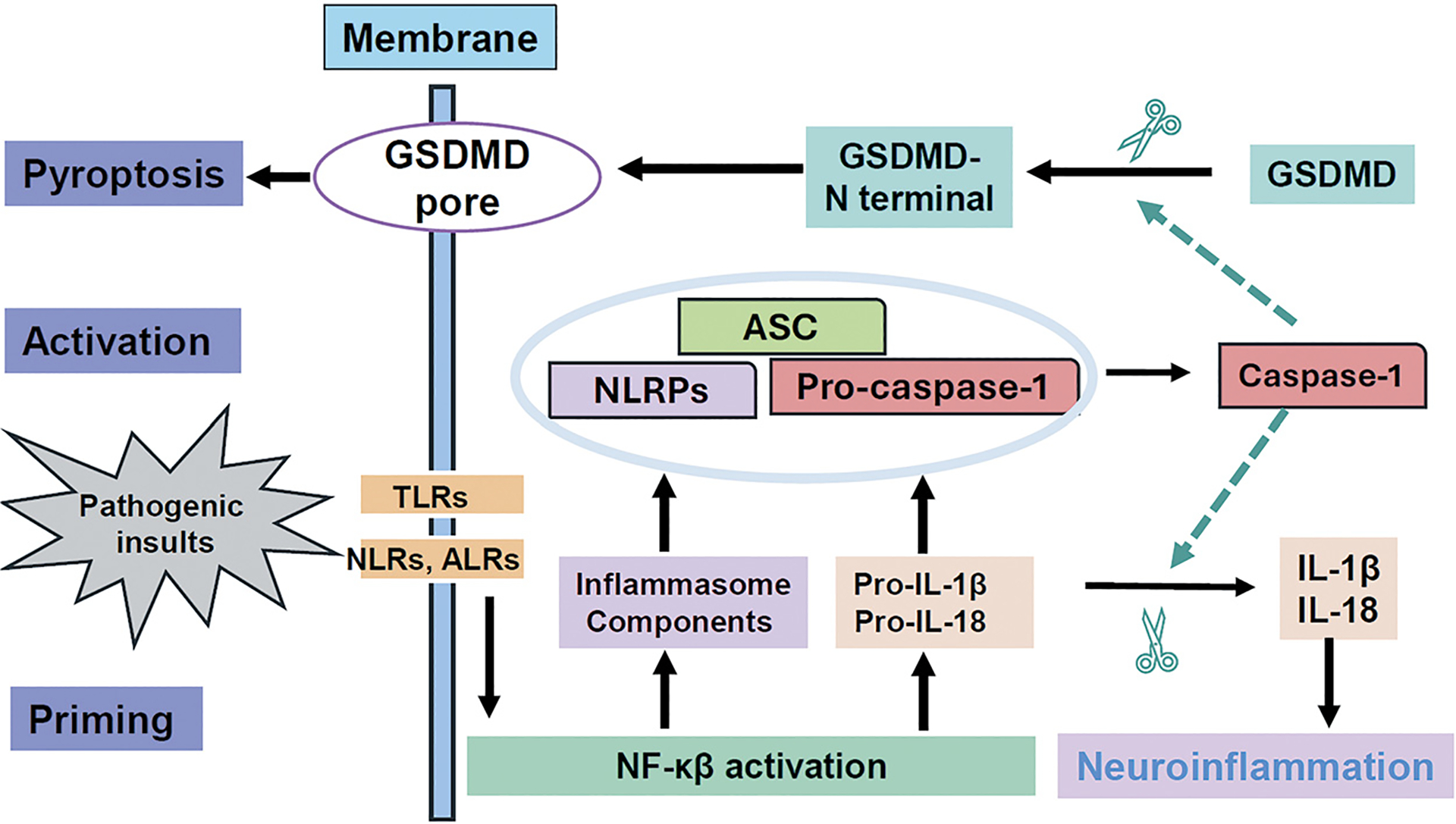
Inflammasome Pathway Activation in the Brain **Step 1, priming stage:** Various pathogenic insults activate PRRs (e.g., TLRs, NLRs, ALRs) and induce NF-κβ pathway activation, resulting in production of inflammasome component proteins (NLRPs, pro-IL-1β, pro-IL-18). **Step 2, activation stage:** The protein complex of NLRP, ASC and pro-IL-1β/pro-IL-18 induces ASC oligomerization, forming ASC specks, and pro-caspase-1 auto-cleavage into matured caspase-1. Caspase-1 then cleaves: 1) pro-IL-1β and pro-IL-18 into IL-1β and IL-18, leading to neuroinflammation; 2) GSDMD to generate N-terminal fragments that translocate to the cell membrane, forming a pore and resulting in pyroptosis.

Inflammasome activation proceeds through a two-step mechanism, see in Figure 2 [2]. Step 1, the priming stage, involves stimuli triggering membrane-bound PRRs (e.g., TLRs and C-type lectin receptors (CLRs)), inducing expression of NLRPs and pro-IL-1β/pro-IL-18 precursor proteins via NF-kB/CREB, AP1, IRF3 and IRF7 signaling pathways to prepare for the inflammasome complex formation. Different stimuli activate distinct PRRs. For example, lipopeptides and glycolipids activate TLR1, TLR2, TLR5 or TLR6; bacterial lipopolysaccharides (LPS) activate TLR4; single-stranded DNA activates TLR7 or TLR8, double-stranded DNA activates TLR3; and CpG DNA activates TLR9. In pathological conditions, amyloid-β (Aβ) and tau (key players in Alzheimer’s’ disease (AD)) induce TLR4 activation [23–37], while α-synuclein (a key player in Parkinson’s disease (PD)) induces TLR2 and TLR5 activation [38,39]. Step 2, the activation stage, an activation signal is essential for the assembly of the inflammasome complex, leading to activation of caspases including caspase-1 and caspase-11 in mouse, or caspase-1, caspase-4, and caspase-5 in human. These activated caspases cleave pro-IL-1β and pro-IL-18 to IL-1β and IL-18 and gasdermin D (GSDMD), releasing its N-terminal pore-forming domain. This domain oligomerizes and inserts in the plasma membrane, forming a pore that results in cytosolic content leakage [41–44] and pyroptosis, a rapid form cell death [2]. Inflammasome-dependent pyroptosis shares features of both apoptosis and necrosis, but it is tightly regulated by distinct signaling pathways. GSDMD controls inflammasome-driven pathology in multiple diseases [41–44]. Thus, inflammasome activation not only upregulates the innate immune response, releasing cytokines that contribute to neuroinflammation, but also induces inflammasome-linked pyroptosis, potentially contributing to neurodegeneration [40].

#### 1.3. Dysregulation of the Inflammasome Pathway and Neurodegenerative Diseases

Chronic inflammasome pathway dysregulation is associated with several auto-inflammatory and autoimmune diseases [45]. Inflammasome activation in CNS has implications for neurodegenerative disease onset and progression [2]. The core pathogenesis of neurodegenerative diseases is believed to involve multiple cellular dysfunctions, including mitochondrial dysfunction, lysosomal destabilization, DNA damage, cytoskeleton abnormalities, neural transport impairment and ionic imbalances [13]. These pathological processes induce abnormal accumulation of toxic proteins, toxins, oxidative species, and damaged DNAs, which acts as stimuli for inflammation and/or neurodegeneration. External environmental risk factors, such as exogenous mitochondrial toxins (rotenone [46,47], MPTP [48]), bacterial and viral infections (e.g., COVID-19), lysosomal destabilization-lysosomotropic detergents (e.g., L-leucyl-L-leucine methyl ester [49]), and some drugs (e.g., nigericin) [26], can also trigger the inflammasome pathway. Increasing evidence implicates inflammasome activation in the pathogenesis of AD, PD, Huntington’s disease (HD), amyotrophic lateral sclerosis (ALS), multiple sclerosis (MS), stroke and other brain disorders [2] (Table 2).

### 2. THERAPEUTIC PERSPECTIVES ON TARGETING THE INFLAMMASOME PATHWAY

Given the critical pathological role of inflammasome pathway in neurodegenerative diseases, targeting this pathway offers a promising therapeutic strategy. The following sections review current approaches, their potential clinical applications, and limitations. To date, four main strategies are being explored for inflammasome pathway regulation and potential therapeutic interventions.

#### 2.1. Small molecule inhibitors

Small molecule inhibitors targeting specific inflammasome components, such as NLRP3, have been tested for their ability to block inflammasome activation in brain disorders (Table 3).

##### A. Inhibitors of NLRP3:

NLRP3 inflammasome is the major inflammasome pathway in neurons and microglia and its activation has implications in various neurodegenerative diseases, including AD, PD, HD, and MS, prion disease and other neuroinflammation-related disorders like brain stroke and injury [2]. There are two types of small molecule NLRP3 inhibitors: direct NLRP3 inhibitors, which bind to specific NLRP3 sites and block inflammasome formation, and indirect inhibitors, which target upstream or downstream pathway components. Among these, only few NLRP3 or caspase-1 inhibitors have entered clinical trials, including MCC950 [50], RP-1127, and glyburide [51] (for stroke by EudraCT 2017-004854-41, ClinicalTrials.gov: NCT01268683 and for traumatic brain injury (TBI), ClinicalTrials.gov: NCT01454154). The caspase-1 inhibitor VX-765 reached Phase II clinical trials for epilepsy (ClinicalTrials.gov: NCT01048255) and psoriasis (ClinicalTrials.gov: NCT00205465), but further development was discontinued. The selective NLRP3 inhibitor RRx-001 is currently in Phase III trial for lung cancer [52], and may have potential for neurodegenerative diseases treatment.

###### A1. Direct NLRP3 Inhibitors.

MCC950 effectively blocks NLRP3-inflammasome pathway activation in *in vitro* and *in vivo* models of AD, PD, TBI, and brain stroke [38,39,53–57]. While currently in clinical trials, further research is needed to establish its safety and efficacy in humans. RRx-001 selectively inhibits NLRP3, currently in a Phase III trial for the treatment of lung cancer [58,59]. Its ability to penetrate the blood–brain barrier (BBB) suggests potential for blocking inflammasome-induced neurodegeneration. Other direct NLRP3 inhibitors include JC-124, sulfonamide compound sulfa 4, NLRP3-inhibitory compound 7, and DAPPD [27–29,32,33,35–37,60]. These have been reported to decrease inflammasome components levels, reduce neuroinflammation and Aβ plaques and improve cognitive impairment in AD mouse models by directly blocking NLRP3 activity [27–29,32,33,35–37,60].

###### A2. Indirect NLRP3 Inhibitors.

Glyburide, a common drug for type 2 diabetes (T2D) treatment, was the first identified NLRP3 inhibitor, though it does not inhibit NLRP1 or NLRC4-dependent IL-1β production [61]. Glyburide also inhibits ATP-, nigericin-, and islet amyloid polypeptide (IAPP)-induced NLRP3-inflammasome activation. Its mechanism of action remains incompletely understood but may be through regulating P2X7 receptor function [61]. Nucleoside reverse transcriptase inhibitors (NRTIs), commonly used to block retrovirus replication, have shown to inhibit NLRP3 activity by targeting P2X7 signaling [62,63]. NRTIs have demonstrated efficacy in mouse models of several inflammatory and autoimmune diseases [62,63]. JNJ-47965567 inhibits the NLRP3 pathway by targeting the P2X7 receptor in a seizure model [64].

Other indirect NLRP3 inhibitors include thioredoxin-interacting protein (TXNIP, upstream of NLRP3), inhibitor, verapamil [64]; caspase-1(downstream of NLRP3) inhibitors: VX-765, VX740, Ac-YVAD-CMK, and CZL80; GSDMD inhibitors: disulfiram, C202-2729, teriflunomide, and necrosulfonamide [65]; NF-κB inhibitors: parthenolide, and BAY11-7082[66]; fenamate classes of non-steroidal anti-inflammatory drugs (NSAIDs); and others: flufenamic and mefenamic acid (inhibiting NLRP3 by blocking volume-regulated anion channels (VRACs))[67], valproic acid, furosemide, and pilocarpine [68]. In addition, β-hydroxybutyrate (BHB) inhibits NLRP3 but lacks specificity as its also inhibits histone deacetylases (HDACs) [69,70]. OLT-1177 (known as dapansutrile), JC-171, Cy09, FC11a-2, 3,4-methylenedioxy-b-nitrostyrene (MNS), acrylate and acrylamide derivatives (e.g., IFN58, IFN39), NBC13, CP-412,245, CP-424,17, CRID1, CRID2, 16673-34-0, C-176, ADU-S100 (antagonist of STING (Stimulator of Interferon Genes)), HC-067047 (TRPV4 channel blocker) [65], NU9056 (histone acetyltransferase inhibitor)[71], and interferon-β (IFNβ) also inhibit NLRP3-inflammasome pathway at some extent [72].

##### B. Strategies Targeting IL-1 (Downstream of NLRP3):

Anakinra (IL-1 receptor antagonist), canakinumab (IL-1β neutralizing antibody), and rilonacept (soluble decoy receptor for IL-1β and IL-1α) have been reported to prevent inflammasome-induced pyroptosis and IL-18-driven immune response [73–81].

##### C. Inhibitors of GSDMD (Downstream of NLRP3):

GSDMD inhibition is another therapeutic strategy to reduce GSDMD-dependent pyroptosis and to prevent inflammasome-driven pathology. GSDMD inhibitors include disulfiram (used to treat alcohol addiction), C202-2729, teriflunomide, and necrosulfonamide, which inhibits GSDMD pore formation, pyroptosis and IL-1β release in cell and mouse models [42,82–84].

##### D. Inhibitors of NLRP1, NLRC4 or AIM2:

Inhibitors targeting NLRP1, NLRC4 or AIM2 include parthenolide, Bay 11–708134, CRID3, auranofin, isoliquiritigenin, 3,4-methylenedioxy-β-nitrostyrene, cyclopentenone prostaglandin and a histone deacetylase 3 (HDAC3) inhibitor RGFP966 [65]. Compounds targeting ASC have not yet been reported.

#### 2.2. Antibodies

A few antibodies targeting inflammasome components and pathway molecules have been developed and tested for their ability to block the inflammasome pathway. These include antibodies against NLRP proteins (NLRP1, NLRP3), ASC (IC100) [85], IL-1β, and IL-18 [65,86]. However, antibody treatment faces challenges, including frequent subcutaneous administration, poor BBB penetration with low CSF concentration, constitutive IL-1β signaling neutralization, and potential increasing infections [87,88].

#### 2.3. RNA Therapy and Gene Deletion

RNA interference (RNAi) has been used to target genes encoding inflammasome components, resulting in knockdown of inflammasome components and attenuate inflammasome activation, neuroinflammation, and neurodegeneration. Small interfering RNAs (siRNAs) targeting NLRP1, NLRP3, and caspase-1 have shown promise in models of neurodegenerative diseases [7,89]. MicroRNAs (miRNAs) also regulate inflammasome components, such miR-9a-5p overexpression inhibiting NLRP1 [90] and miR-223–3p overexpression reducing NLRP3 [91]. Gene deletion of inflammasome components or pathway molecules (*Nlrp1−/−* [92,93], *Nlrp3−/−* [23], *Nlrc4−/−*[94], *Nlrp−/−*[95], *Aim2−/−* [96,97], *Casp1−/−*[98], *Asc−/−* [99], *IL18−/−*[100], and *Il1r1−/−* [101]) has been reported to reduce neuroinflammation and neurodegeneration.

#### 2.4. Lifestyle Interventions

Lifestyle interventions, including diet, stress management, and exercise, have been shown to reduce inflammasome activity and neurodegenerative disease risk. Studies suggest that the gut–brain axis may influence early neuroinflammation priming events [102]. A chronic Western diet induces NLRP3 activation, suggesting that healthy food choices may increase endogenous inhibitory of NLRP3 inflammasome[69,70]. Intermittent fasting increases levels of BHB (an endogenous NLRP3 inhibitor) in mice [69,70]. A tryptophan-rich diet increases indoles and reduces neuroinflammation in amyloid precursor protein/presenilin-1 (APP/PS1) mice [103]. Probiotics, as dietary supplements, reduce inflammasome activation in the central nervous system in mice [102]. Healthy sleep increases nobiletin, and a healthy circulatory system increases angiotensin [104]. These molecules reduce NLRP3-inflammasome activation and IL-1β levels in the brain, rescuing cognitive impairment in AD models [103,105]. In PD studies, gut microbiota changes produce urolithin and BHB, which negatively regulate NLRP3 and slow PD progression [69,70]. Dietary vitamin C and D, and perillyl alcohol effectively reduce brain NLRP3 levels and improve motor impairment in PD mouse models [106–108]. In MS mouse models, dimethyl fumarate (a glycolysis intermediate) and nicotinamide adenine dinucleotide (NAD+) reduce NLRP3 and IL-1β [109,110]. Ghrelin, a gut hormone activating glucagon-like peptide 1 receptor, reduces NLRP3 inflammasome pathway and rescues MS-like phenotypes [111]. In epilepsy mouse models, dietary ω fatty acids (eicosapentaenoic acid and docosahexaenoic acid) reduce inflammasome activation and attenuate seizure [112,113]. Dietary ω3 fatty acids or saffron extract reduces TBI-induced inflammasome activation and inflammatory damage in mice [114,115].

#### 2.5. Limitations

While small molecule inhibitors, antibodies and genetic alterations targeting inflammasome pathway have been tested in disease models, few have reached human clinical trials. Despite promising results from *in vitro* and animal studies, these strategies have limitations requiring further investigation to determine safety and efficacy to humans. These limitations include safety concerns due to uncertain inhibitor specificity, limited brain efficacy due to poor BBB penetration, the high costs associated with gene therapy, and an incomplete understanding of how inflammasomes contribute to neurodegeneration. Safety criteria for targeting inflammasomes have not yet been established. It remains unknown whether these approaches induce downregulation of the innate immune system, increasing susceptibility to infections with long-term treatment, and whether they are safe and well-tolerated in humans. Developing new treatments for neurodegenerative diseases is complex and expensive, particularly for antibody and gene therapies. Crucially, the mechanisms by which inflammasomes contribute to neurodegeneration are not fully understood, necessitating further research to identify precise targets and affective disease-specific treatment strategies.

## CONCLUSION

Targeting inflammasomes holds promise for developing new treatments for neurodegenerative disorders, including AD, PD, HD, ALS, MS, TBI and brain stroke. However, significative challenges remain in developing safe, effective, and specific treatments with minimal side effects. Further research is needed to dissect the complex molecular mechanisms of the inflammasome pathway in the innate immune system and the CNS. A deeper understanding of the pathophysiological roles of the inflammasome pathway will aid in identifying precise and effective therapeutic targets and intervention strategies for neurodegenerative diseases and other related brain disorders.

## Figures and Tables

**Table 1.** Pathogenic Insults Activating Inflammasomes in the Brain

Brain disorders	Pathogenic Insults
Alzheimer’s disease	Aβ1–42
	Hyperphosphorylated tau
Parkinson’s disease	α-synuclein
	Impaired mitophagy
	Toxins, MPTP, rotenone
Amyotrophic lateral sclerosis	Mutant SOD1
	TDP43 aggregates
Huntington’s debases	Mutant huntingtin
Multiple sclerosis	DAMPs resulting from T-cell-mediated myelin damage
Traumatic brain injury	DAMPs resulting from blunt force or penetrating injury
Stroke	DAMPs resulting from ischemic or hemorrhagic injury
Epilepsy	DAMPs resulting from mild seizure-related hemorrhages
Other brain disorders	DAMPs or PAMPs resulting from pathogen-triggered neuroinflammation

**Table 2.** Dysfunction of Inflammasome Pathway in Neurodegenerative Diseases and Other Brain Disorders

Pathogenic insult	Inflammasome activation	Model system	Treatment
Aβ	TLR4 [116]	Neurons [116]	TLR4 antagonists RsLA, but not TAK-242 [116]
	NLRP3 [23–37]	Microglia [23,24,117]	NLRP3-KO [23], Verapamil (TXNIP) channel [24]
	ASC [36,117,118]	Astrocytes (5xFAD) [34,119]	ASC-KO [119]
	Caspase-1 [36]	PBMC [25] [31]	Stavudine (D4T) [25]
	IL-1β [119]	PC12 [35]	AAV9-siRNA-caspase-1 [26]
	NLRP1 [118] [120]	BV2 [36,37]	VX-765 [27]
	NLRP2 [34]	Human post-mortem AD brains [118] [121,122]	OLT1177 [28]
		APP/PS1 mice [26–29,33]	DAPPD [29]
		3xTg-AD [30]	JC-124 [60]
		5xFAD [31]	Eriodictyol and Homoeriodictyol [32]
		Tg-CRND8 mice [60]	Chemerin-9 [33]
		Aβ injection [32]	Emodin [35]
			Dedicator of cytokinesis 8 (DOCK8) [36]
			Salvianolic acid B [37]
α-synuclein	NLRP3	A53T-viral-α-synuclein, PD human brains [38,39]	MCC950 [38,39]
		α-synuclein PFF [123]	ASC-KD [123]
		*In vitro* α-synuclein oligomer [124]	Glycation [124]
		BV2, MPTP mice [125]	Echinacoside [125]
Parkin	NLRP3	Parkin−/−, α-synuclein PFF, human PD postmortem DA neurons	NLRP3-shRNA [126]
MPTP	NLRP3	MPTP injected mice	Glia maturation factor -KO [127]
			NLRP3-KO [128]
			hepatic Nlrp3 inhibition by Nlrp3-siRNA-Lentivirus [129]
			MCC950 [48]
			Indole derivative NC009-1 [130]
			OLT1177 [131]
Rotenone	NLRP3	Primary mouse microglia, chronic rotenone rodents [46]	Canagliflozin [47]
		Rotenone rats [47]	
Paraquat and Maneb	NLRP3	Paraquat and Maneb treated mice	CD11b-KO [132]
			Glybenclamide [133]
Mutant huntingtin	NLRP3 [134–137]	R6/2 [134,136,137]	MCC950 [134]
	Caspase 1 [136]	Hdh150Q [135]	Gal3 inhibitor [135]
		HD human brains [135]	Olaparib (PARP-1 Inhibitor) [136]
SOD1	NLRP1,NLRC4 [138]	G93A mice, ALS human brain [138]	MCC950 [142,143]
	NLRP3	Astrocytes [139] G93A [140–142]	
TDP-43	NLRP3	Q331K mice [142,144], microglia [145]	MCC950 [142]
Cuprizone	Caspase-1 [146] [147]	MS human brains [146]	VX-765 [146]
	NLRP3 [94,146–153]	Cuprizone-exposed mice [94,146–153]	Nebivolol [147]
	NLRC4 [94]		Progesterone [149]
			TGN020 (Aquaporin 4 blocker) [151]
			Prednisone [152]
			17β-Estradiol [153]
			Nlrp3-KO, Nlrc4-KO [94]
MS	NLRP3 [66,109,154–162]	EAE mice [66,85,109,110,154–161,163]	MCC950 [154]
	IL-18 [154,162]	Relapsing-remitting MS patients [162]	1,2,4-Trimethoxybenzene [155]
	ASC [85,155]		Amifostine (radioprotective drug) [156]
	GASDMD [110,163]		AZD8055 (autophagy activator) [157]
			Bixin (carotenoid) [158]
			Ellagic acid [159]
			OLT1177 [160]
			C202-2729 [163]
			BAY11-7082 (NF-kappaB blocker) [66]
			IC100 (anti-ASC monoclonal antibody) [85]
			Succination [110]
			Nicotinamide adenine dinucleotide [109]
			Ghrelin [161]
Lysolecithin	NLRP3[164]	Lysolecithin mice [91,164]	miR-223 [91,164]
		Macrophages and microglia [164]	MCC950 [164]
Amygdala kindling	NLRP1 [165]	Amygdala kindling-induced rats [89,165]	NLRP1-KD [165]
	Caspase-1 [89,165]		Caspase-1-KD [89,165]
	NLRP3 [89]		NLRP3-KD [89]
Kainic acid (KA)	NLRP3 [112,166–169]	KA-induced epileptic rats [166,168]	Huperzine A [166]
	Caspase-1 [112,166]	KA-induced aggregation of Aβ mice [167]	Valproic acid and furosemide combination [168]
	IL-1β [112]	KA-induced temporal lobe epileptic mice [112,169]	GPR120 [112]
	NLRP1 [168]		VX765 [112]
Pentylenetetrazole (PTZ)	NLRP3 [113,170–172]	PTZ-Induced epileptic mice [113,170,172]	Eicosapentaenoic acid (EPA) and docosahexaenoic acid (DHA) [113]
	ASC [113,170]	PTZ-Induced epileptic rats [171]	
	Caspase-1 [113]		Sinapic Acid [170]
	IL-1β [170]		Ibuprofen [171]
	IL-18 [171]		CY-09 [172]
Pilocarpine	NLRP3 [40,68,173,174]	Pilocarpine-induced status epileptic mice [40,68,173]	MCC950 [40,173]
	IL-1 [174]	TLE patients’ brain [173]	SR9009 (Rev-Erbalpha agonist) [68]
		Status epilepticus patients’ brain [174]	Anakinra/canakinumab (IL-1 blocker) [174]
TBI	NLRP3 [53,54,175]	Controlled cortical impact injury mice [53,175,177,178]	MCC950 [53] [54]
	ASC [175–178]		Oridonin [176]
	Caspase-1 [176–178]	Closed-head injury model [54,176]	GSDMD-KO [179]
	GSDMD [177–179]		NLRP3 -KO [179]
	IL-1 [175]		JC124 [175]
	IL-18 [177,178]		VX765 [177]
			Ac-YVAD-cmk [178]
Stroke	NLRP1 [90,180,181]	Intracerebral hemorrhage [56,180,186,187,189,190]	CCR5 [180]
	ASC [180,182]	Focal cerebral ischemia [55,181,184,185,188]	NLRP2 siRNA [185]
	Caspase-1 [180,183,184]	Photothrombotic single stroke mice [182,183]	Immunoglobulin [181]
	IL-18 [180,181]	Middle cerebral artery occlusion [90,95–97,191,192]	BRCC3 siRNA [191]
	IL-1β [180,181,183]	Human stroke postmortem brains [181]	NLRP10-KO [95]
	GSDMD [180,183]		AIM2-KO [96,97]
	NLRP2 [185]		VX-765 [183]
	NLRP3 [55,56,181,182,186–190]		NLRP3 siRNA [186,188]
			RGFP966 (HDAC3 inhibitor) [192]
	NLRP6 [191]		miR-9a-5p [90]
	NLRP10 [95]		MCC950 [55,56]
	TLR4 [95]		Edaravone [190]
	AIM2 [96,97,192]		CZL80 [184]

**Note: MPTP:** N-methyl-4-phenyl-1,2,3,6-tetrahydropyridine; **KO:** knockout; **PBMC:** peripheral blood mononuclear cells; **PC12:** rat cell line; **BV2:** immortalized microglial cell line; **3xTg-AD:** AD mouse model; **5xFAD:** AD mouse model; **Tg:** transgenic; **AAV9:** adeno-associated virus serotype 9; **DA:** dopaminergic neurons; **PFF:** preformed fibrils; **shRNA:** small hairpin RNA, **EAE:** MS mouse model; **miR:** micro-RNA; **KA:** kainic acid; **PTZ:** pentylenetetrazole.

**Table 3.** Promising Compounds Targeting Inflammasome Pathway for Therapeutics

Strategy	Compound	Effect/Application
Direct NLRP3 inhibitors	MCC950	Phase I clinical trial
	RRx-001	Phase III trial for lung cancer
	JC-124	
Indirect NLRP3 inhibitors	Glyburide	Common use for T2D
	JNJ-47965567	Altering P2X7 signaling
	NRTIs	Blocking VRACs
	TXNIP inhibitor	Inhibit HDACs
	NSAIDs	Acrylate and acrylamide derivatives:
	Flufenamic and mefenamic acid	No report *in vivo* testing
	BHB	STING antagonist
	OLT-1177	Blocking the TRPV4 channel
	JC-171,	Histone acetyltransferase inhibitor
	Cy09 and FC11a-2, MNS	
	IFN58, IFN39, NBC13	
	CP-412,245 and CP-424,174	
	CRID1 and CRID2	
	6673-34-0	
	C-176	
	ADU-S100	
	HC-067047	
	NU9056	
	Interferonβ	
	Fc11a-2	
	CY-09	
NF-κB inhibitors	Parthenolide	
	BAY11-7082	
Caspase-1 inhibitors	Vx-765	No further development after phase II clinical trial
	Vx-740	
	Ac-YVAD-CMK	
	CZL80	
Targeting IL-1	Anakinra	IL-1 receptor antagonist
	Canakinumab	IL-1β neutralizing antibody
	Rilonacept	Soluble decoy receptor for IL-1β and IL-1α
Targeting GSDMD	Disulfiram	Common use for alcohol addiction treatment
	C202-2729	
	Teriflunomide	
	Necrosulfonamide	
Targeting NLRP1, NLRC4 or AIM2	Parthenolide	Histone deacetylase 3 (HDAC3) inhibitor
	CRID3	
	Auranofin	
	Isoliquiritigenin	
	3,4-methylenedioxy-β-Nitrostyrene	
	Cyclopentenone prostaglandin RGFP966	

## List of abbreviations:

CNS: Central nervous system
TNFs: Tumor necrosis factors
IL: interleukin
CSF: Cerebrospinal fluid
NLRP: Nucleotide-binding oligomerization domain, Leucine rich Repeat and Pyrin domain containing
CARD: Caspase recruitment domain
ASC: Apoptosis-associated speck-like protein containing CARD
PRRs: Pattern recognition receptors
PAMPs: Pathogen-associated molecular patterns
DAMPs: Damage-associated molecular patterns
NLRs: Nod-like receptors
TLRs: Toll-like receptors
ALRs: Absent in melanoma 2-like receptors
IFI16: Interferon-γ-inducible protein 16
ATP: Adenosine triphosphate
PYD: Pyrin domain
LRR: Leucine-rich repeat domain
NACHT/NBD: Nucleotide-binding and protein oligomerization domain
CLRs: C-type lectin receptors
NF-κB: Nuclear factor-κB
CREB: cAMP response element binding protein
AP-1: Activator protein 1
IRF: Interferon regulatory factor
Aβ: Amyloid-β
AD: Alzheimer’s’ disease
PD: Parkinson’s disease
CLRs: C-type lectin receptors
LPS: Lipopolysaccharides
GSDMD: Gasdermin D
HD: Huntington’s disease
ALS: Amyotrophic lateral sclerosis
MS: Multiple sclerosis
TBI: Traumatic brain injury
BBB: Blood brain barrier
T2D: Type 2 diabetes
IAPP: Islet amyloid polypeptide
NRTIs: Nucleoside reverse transcriptase inhibitors
TXNIP: Thioredoxin-interacting protein
NSAIDs: Non-steroidal anti-inflammatory drugs
VRACs: Volume-regulated anion channels
BHB: β-hydroxybutyrate
HDACs: Histone deacetylases
MNS: 3,4-methylenedioxy-b-nitrostyrene
STING: Stimulator of interferon genes
IFNβ: Interferon-β
RNAi: RNA interference
siRNA: Small interfering RNA
miRNAs: microRNAs
APP/PS1: Amyloid precursor protein/presenilin-1
NAD+: Adenine dinucleotide
